# Population genetic structure of eelgrass (*Zostera marina*) on the Korean coast: Current status and conservation implications for future management

**DOI:** 10.1371/journal.pone.0174105

**Published:** 2017-03-21

**Authors:** Jae Hwan Kim, Ji Hyoun Kang, Ji Eun Jang, Sun Kyeong Choi, Min Ji Kim, Sang Rul Park, Hyuk Je Lee

**Affiliations:** 1 Molecular Ecology and Evolution Laboratory, Department of Biological Science, College of Science and Engineering, Sangji University, Wonju, Republic of Korea; 2 Korean Entomological Institute, Korea University, Seoul, Republic of Korea; 3 Estuarine and Costal Ecology Laboratory, Department of Marine Life Sciences, Jeju National University, Jeju, Republic of Korea; National Cheng Kung University, TAIWAN

## Abstract

Seagrasses provide numerous ecosystem services for coastal and estuarine environments, such as nursery functions, erosion protection, pollution filtration, and carbon sequestration. *Zostera marina* (common name “eelgrass”) is one of the seagrass bed-forming species distributed widely in the northern hemisphere, including the Korean Peninsula. Recently, however, there has been a drastic decline in the population size of *Z*. *marina* worldwide, including Korea. We examined the current population genetic status of this species on the southern coast of Korea by estimating the levels of genetic diversity and genetic structure of 10 geographic populations using eight nuclear microsatellite markers. The level of genetic diversity was found to be significantly lower for populations on Jeju Island [mean allelic richness (AR) = 1.92, clonal diversity (*R*) = 0.51], which is located approximately 155 km off the southernmost region of the Korean Peninsula, than for those in the South Sea (mean AR = 2.69, *R* = 0.82), which is on the southern coast of the mainland. South Korean eelgrass populations were substantially genetically divergent from one another (*F*_ST_ = 0.061–0.573), suggesting that limited contemporary gene flow has been taking place among populations. We also found weak but detectable temporal variation in genetic structure within a site over 10 years. In additional depth comparisons, statistically significant genetic differentiation was observed between shallow (or middle) and deep zones in two of three sites tested. Depleted genetic diversity, small effective population sizes (*N*_e_) and limited connectivity for populations on Jeju Island indicate that these populations may be vulnerable to local extinction under changing environmental conditions, especially given that Jeju Island is one of the fastest warming regions around the world. Overall, our work will inform conservation and restoration efforts, including transplantation for eelgrass populations at the southern tip of the Korean Peninsula, for this ecologically important species.

## Introduction

Seagrasses, marine angiosperms, play a pivotal role in ecosystem functioning and services in coastal zones. For example, they are primary producers and seagrass beds also provide important habitats, serving as both nursery and grazing areas for other marine organisms [1–3]. They are often called “ecosystem engineers” as they can modify surrounding biotic and abiotic marine environments, creating their own habitats. The structural components of seagrass leaves, rhizomes, and roots alter water currents, buffer physical forces of waves [4], and filter organic nutrients or pollutants, which help to improve water quality [5], stabilize sediment bottoms [6], and enhance carbon sequestration [7–9]. Seagrasses are thus considered a valuable ecosystem component in coastal and estuarine habitats [10].

Unfortunately, seagrass populations have been disappearing recently worldwide, primarily due to anthropogenic pressure such as reclamation, dredging, and climate change [11,12]. According to a recent meta-analysis of quantitative data on seagrass coverage from 215 sites around the world, more than 51,000 km^2^ of seagrass meadows have been lost during the past 127 years [11]. The rate of seagrass decline has accelerated from a median of 0.9% per year before 1940 to 7% per year since 1990. Therefore, seagrass meadows are now regarded as the most threatened ecosystem on earth amongst all coastal ecosystems (e.g., mangroves, coral reefs, and tropical rainforests) [11].

*Zostera marina* (common name “eelgrass”), which is the most wide-ranging seagrass species in the northern hemisphere, including the North Atlantic and North Pacific Oceans, is also the predominant seagrass species along the Korean coasts [13,14]. This species usually occurs from intertidal to subtidal areas (e.g., it typically occurs at depths of 1–7 m relative to the mean low tide point on the southern coast of Korea) [14].

Understanding the extent of intraspecific genetic diversity and population genetic structure of *Z*. *marina* provides important information for monitoring and conservation or restoration efforts of seagrass meadows [15–17]. Population genetics surveys allow for inferring the population demographic history of *Z*. *marina* (e.g., population bottleneck), assessing population connectivity (i.e., contemporary gene flow), which is especially important for determining the size of a management unit, and gauging the likelihood of population persistence and adaptive potential in response to anthropogenic pressure such as climate change [16]. A number of population genetics studies of *Z*. *marina* have been conducted to examine spatial and temporal variation in the population genetic structure as well as clonal patch dynamics [18–20]. The observed patterns of the genetic diversity and population connectivity of this species, however, appear to vary among coastal regions and also with sampling or geographic scales examined [21].

Information on genetic variation/diversity permits testing whether the current population has lost genetic variability through the effects of genetic drift, particularly when the target population is isolated from surrounding populations [22,23]. Genetic diversity is well known to play a significant role in the ecological performance of natural seagrass populations [24,25], and it therefore strongly affects the ultimate outcome of conservation and restoration efforts of seagrasses [17,26]. Enriched within-population genetic diversity safeguards an increase in seagrass population density and biomass, enhances coexisting faunal abundance through community-level positive feedback [25], ensures rapid recovery after disturbance events (e.g., geese grazing) [24], and helps withstand biotic and abiotic environmental changes [27]. As a consequence, taking into account the information on population genetic structure of *Z*. *marina* helps facilitate effective population restoration and management plans [17,26].

*Z*. *marina* occasionally displays complex reproductive strategies associated with environmental conditions, evolving divergent life history tactics [28,29]. Populations of *Z*. *marina* separated by water depth within the same locality have recently been suggested to adapt to different light conditions, evolving alternative life history strategies (e.g., annual or perennial life histories) [29]. However, how eelgrass populations have evolved different reproductive strategies along the depth gradient and whether these depth populations within a site share a similar genetic makeup or distinct genetic clusters remain unresolved. Nevertheless, a recent study found some detectable genetic divergence between shallow and deep zones of *Z*. *marina* in San Francisco Bay, California, USA [28]. Further studies are required to test the hypothesis that ecologically divergent populations of *Z*. *marina* isolated by depth also comprise different genetic sub-populations rather than a single gene pool.

In recent years, a number of studies on Korean populations of *Z*. *marina* have been carried out, focusing on their ecological and physiological characteristics such as distribution patterns, growth dynamics, recruitment, and photosynthetic capability [30–34]. Additionally, several transplantation projects have been successfully conducted for seagrass habitat restoration [35–38]. Although studies have highlighted the significant role of genetic diversity in the ecological performance of seagrass populations [24,25], little effort has been made to understand the genetic structure and genetic diversity in *Z*. *marina* populations in Korea for the purpose of their conservation and restoration efforts.

In the present study, we examined the level of genetic diversity and the population genetic structure of *Z*. *marina* on the southern coast of Korea (including Jeju Island which is located approximately 155 km off the southernmost region of the mainland) to assess the current population genetic status and conservation necessity. We also tested whether ecologically divergent populations by water depth within three localities differ in genetic composition. The specific objectives of this study were to (1) examine and compare the levels of within-population genetic diversity in *Z*. *marina* between five populations in Jeju Island and five populations in the South Sea on the southern coast of Korea; (2) examine the spatial genetic structure on different geographical scales; (3) test whether there was a change in genetic composition over a 10 year-period using temporal samples; and (4) investigate whether there was significant variation in the population structure between shallow and deep populations within each of three sites. The results of our study will provide a basic but significant guideline for designing effective management, conservation, and restoration plans of this ecologically crucial species, *Z*. *marina*, on the Korean coast.

## Materials and methods

### Sample collection

**Fig 1:**
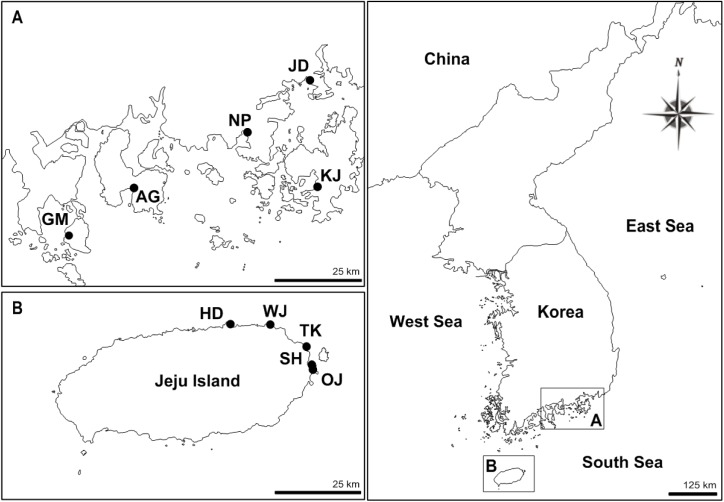
Sampling sites of *Zostera marina* in the South Sea on the southern coast of Korea and in Jeju Island at the southern tip of Korea. (A) South Sea populations: Gamak Bay (GM, 34˚37’55”N; 127˚43’21”E), Aenggang Bay (AG, 34°46’30”N; 127°56’34”E), Nampo Port (NP, 34°57’25”N; 128°19’20”E), Jindong Bay (JD, 35°6’38”N; 128°32’41”E), and Koje Bay (KJ, 34°48’7”N; 128°35’7”E); (B) Jeju Island populations: Hamdeok (HD, 33°33’00”N; 126°39’24”E), Woljeong (WJ, 33°33’56”N; 126°46’47”E), Tokki-seom (TK, 33°31’24”N; 126°54’04”E), Siheung (SH, 33°28’26”N; 126°55’25”E), and Ojo (OJ, 33°28’05”N; 126°55’30”E).

We sampled 454 individuals of *Z*. *marina* from 10 different localities on the southern coast of Korea (including Jeju Island, which is located off the southern tip of Korea) in August 2015 (Table 1, Fig 1). Sampling sites at Jeju Island included Hamdeok (HD), Tokki-seom (TK), Ojo (OJ), Woljeong (WJ), and Siheung (SH), and those in the South Sea on the southern coast of Korea included Gamak Bay (GM), Jindong Bay (JD), Nampo Port (NP), Aenggang Bay (AG), Koje Bay (KJ) (Table 1, Fig 1). Near WJ in Jeju Island, human-mediated transplantation project was undertaken using other populations as a source material in 2009 [39]. However, their source population was not reported, so it remains unknown. Note that the WJ samples used in this study were obtained from areas that differed from the sites where restoration efforts had been implemented. No specific permission to collect samples was required at the study sites, and the field study did not involve endangered or protected species. Samples were collected in monotypic meadows of *Z*. *marina* by both wading and diving at a distance of 1–2 m intervals between samples using a linear transect to obtain randomly chosen ramets within each location [40]. All the samples were collected at 1–2 m intervals of each other within sites and these sampling distances were kept to be identical among sampling localities. *Z*. *marina* meadows at five sites on Jeju Island were mapped in the field with Global Positioning Systems (OziExplorer program).

**Table 1:** Estimates of genetic diversity at eight microsatellite loci for 16 populations (including temporally replicated and depth samples) of *Zostera marina* in South Korea. *N*_r_: number of ramets sampled, *N*_g_: number of genets in sample, *P*_ID_: probability of identity among multilocus genotypes (MLG), *P*_IDsib_: probability of identity among MLG with high sibling mating, *N*_A_: mean number of alleles per locus, *R*: clonal diversity, AR: allelic richness, *H*_O_: observed heterozygosity, *H*_E_: expected heterozygosity, HWE (*P*): *P* values for multilocus tests for Hardy-Weinberg equilibrium (HWE), *F*_IS_: inbreeding coefficient. Population abbreviations as in text and Fig 1. *: significant after a sequential Bonferroni correction applied (*P* < 0.05); **: *P* < 0.01; NS: not significant.

POPULATION		*N*_r_	*N*_g_	*P*_ID_	*P*_IDsib_	*N*_A_	*R*	AR	*H*_O_	*H*_E_	HWE (*P*)	*F*_IS_
**Jeju Island**	**HD**	30	9	0.0175	0.2215	1.625	0.276	1.524	0.229	0.194	NS	-0.198
	**WJ**	30	7	0.0001	0.0677	2.500	0.207	2.124	0.286	0.341	NS	0.190
	**TK**	30	21	0.0011	0.0529	2.500	0.690	2.000	0.346	0.344	*	-0.006
	**SH**	30	27	0.0002	0.0289	3.125	0.897	2.255	0.304	0.405	**	0.251
	**OJ**	29	14	0.0072	0.1262	1.875	0.464	1.687	0.250	0.256	NS	0.022
**Jeju Island mean**		29.800	15.600	0.0052	0.0994	2.325	0.507	1.918	0.283	0.308	**	0.052
**South Sea**	**GM**	30	27	0.0000	0.0139	4.125	0.897	2.597	0.462	0.482	NS	0.040
	**AG-S**	30	19	0.0000	0.0088	4.250	0.621	2.792	0.507	0.530	NS	0.044
	**AG-M**	18	16	0.0000	0.0100	4.250	0.882	2.795	0.514	0.519	NS	0.010
	**AG-D**	30	17	0.0000	0.0074	3.750	0.552	2.804	0.625	0.556	NS	-0.129
	**NP**	30	30	0.0000	0.0160	5.000	1.000	2.651	0.475	0.452	NS	-0.051
	**JD-S**	30	28	0.0000	0.0177	5.250	0.931	2.721	0.476	0.441	NS	-0.082
	**JD-D**	30	27	0.0000	0.0173	4.500	0.897	2.689	0.425	0.448	*	0.053
	**KJ15-S**	30	27	0.0002	0.0293	4.000	0.897	2.368	0.380	0.404	NS	0.062
	**KJ15-D**	30	22	0.0000	0.0107	4.750	0.724	2.795	0.390	0.512	**	0.243
	**KJ05-S**	18	14	0.0001	0.0314	3.125	0.765	2.358	0.389	0.408	NS	0.047
	**KJ05-D**	29	15	0.0002	0.0372	3.250	0.500	2.296	0.342	0.392	*	0.100
**South Sea mean**^a^		28.667	23.667	0.0000	0.0146	4.431	0.822	2.690	0.473	0.483	NS	0.021
**The grand mean for all populations**		28.375	20.000	0.0017	0.0436	3.617	0.700	2.404	0.400	0.418	**	0.037

^a^: The values for South Sea mean were estimated after the KJ samples obtained in 2005 (KJ05-S, KJ05-D) were excluded.

To test whether there was temporal variation in the population genetic structure of *Z*. *marina* in Koje Bay on the southern coast of Korea, plants were sampled during two separate sampling periods, which were 10 years apart (July 2005 and August 2015) (KJ05 and KJ15). Due to the reported local or fine-grained genetic structure of this species [18,19], we attempted to collect the second samples (i.e., 2015 samples) from exactly the same microhabitats as the previous samples from 2005 in order to rule out a confounding spatial effect. To investigate whether there was significant genetic structure between populations by water depth within sites, three (KJ, JD, and AG) of the five sites were chosen in the South Sea for depth-specific populations, which were collected at both shallow zone (S, water depth ranges from 0 to 0.6 m) and deep zone (D, from 1.6 to 8.5 m). At AG, an additional population was sampled at a middle zone (AG-M, from 1.8 to 2.3 m). Note that the KJ population was collected in the intertidal zone for the shallow population (intertidal zone is always above water at low tide and under water at high tide).

Collected samples were washed using freshwater and raked to remove epiphytic algae using a sterilized razor blade. Leaf samples were dried at 60°C for 24 h and then ground using a TissueLyserII (QIAGEN). Powdered samples were transferred to a 1.5-ml microcentrifuge tube with silica gel and stored at –20°C until genetic analysis.

### DNA extraction, PCR, and microsatellite genotyping

Genomic DNA was extracted from pulverized leaf samples using both i-genomic Plant DNA Extraction Mini Kit (iNtRON Biotechnology) and DNeasy Plant Mini Kit (QIAGEN) according to the manufacturer’s directions. DNA concentration was determined using a Qubit® 2.0 Fluorometer (Invitrogen). Eight microsatellite loci were amplified by polymerase chain reaction (PCR) with the published primers Zosmar GA1, GA2, GA3, GA4, CT3, CT12, CT20, and CT35 [41,42]. Each forward primer was labeled with a fluorescent dye (FAM, VIC, NED, TAMRA, and PET). PCR amplification was accomplished in a reaction volume of 15 μl containing 25 μM of each dNTP (Bio Basic), 0.6 μM each of the forward and reverse primers, 0.2 units of *Taq* DNA polymerase (Thermo Fisher Scientific), 1× PCR buffer, and approximately 5−10 ng of template DNA. PCR cycling conditions comprised an initial denaturation phase at 94°C for 5 min, followed by 37 cycles of 94°C for 20 sec (denaturation), 54−57°C for 30 sec (annealing), and 72°C for 30 sec (extension), followed by a terminal extension phase at 72°C for 12 min in a 2720 thermal cycler (Applied Biosystems). Each PCR product was checked on a 2% agarose gel stained with Redsafe^TM^ (iNtRON Biotechnology). Amplified PCR products were then electrophoresed in an ABI 3730xl automated DNA sequencer (Applied Biosystems). Fragment sizes were compared with that of a ROX 500 bp size standard (ABI) as determined using GeneMapper software v5.0 (Applied Biosystems).

### Statistical analyses

#### Clonal and genetic diversities

To avoid resampling multiple examples of the same clonal individual, we defined an individual plant (genet) as having a unique multi-locus genotype (MLG) using GENALEX v6.5 [43]. Where multiple samples (ramets) shared a single MLG, all but one ramet was removed for further analyses. We calculated clonal diversity *R* = (*G*– 1)/(*N*– 1), where *G* = the number of genets and *N* = the total number of ramets sampled [44]. Higher values of *R* denote lower levels of clonality. Once replicate ramets were removed, we assessed the statistical power of the pruned dataset to detect clones using the probability of identity values, *P*_IDunbiased_ (hereafter *P*_ID_) and *P*_IDsib_ [45] estimated in GIMLET v1.3.3 [46] for each population, and depth and temporal samples.

To evaluate microsatellite diversity in the Korean eelgrass, the mean number of alleles per locus (*N*_A_), observed (*H*_O_) and expected (*H*_E_) heterozygosity, inbreeding coefficient (*F*_IS_) [47], and allelic richness (AR) corrected for unequal sample sizes were calculated using GENEPOP v4.3 [48] and FSTAT v2.9.3.2 [49]. We conducted two separate Mann-Whitney *U* tests to investigate if there were significant differences in the levels of clonal and genetic diversities (e.g., *R*, AR) between populations from Jeju Island (*n* = 5) and from the South Sea (*n* = 9) after the KJ samples obtained in 2005 (KJ05-S, KJ05-D) were excluded (see below). We tested for the presence of null alleles using MICRO-CHECKER v2.2.3 with 1000 randomizations at the 95% confidence level [50]. Genotypes at the eight microsatellite loci were tested for linkage disequilibrium (LD, nonrandom associations of alleles from different loci) using the entirely pooled sample, and multilocus tests for Hardy Weinberg equilibrium (HWE) were undertaken using GENEPOP. The 95% significance levels for every exact test for both LD and HWE were adjusted using a sequential Bonferroni correction.

Evidence of recent population bottlenecks was tested using BOTTLENECK v1.2.02 with the two-phase mutation model (TPM) [51]. A population bottleneck can be identified by the occurrence of a mode-shift (i.e., allele distribution shift) and/or a significant heterozygosity excess tested statistically by a Wilcoxon sign-rank test [52]. Contemporary effective population sizes (*N*_e_) were also calculated for each of the samples based on the LD method in NeEstimator v2.01 [53].

#### Population genetic structure

To evaluate the spatial population genetic structure of *Z*. *marina* on the southern coast of Korea, a hierarchical analysis of molecular variance (AMOVA) was performed in ARLEQUIN v3.5 [54]. The spatial AMOVA was conducted by grouping the 14 populations (Jeju Island: *n* = 5; South Sea: *n* = 9) into two different regions (Jeju Island and South Sea). The Jeju Island region comprised HD, WJ, TK, SH, and OJ, and the South Sea region comprised GM, AG-S, AG-M, AG-D, NP, JD-S, JD-D, KJ15-S, and KJ15-D. The KJ populations sampled in 2005 (KJ05-S, KJ05-D) were excluded from this analysis because detectable temporal variation was observed (see below). To further investigate spatial, temporal, and depth-specific genetic differentiation between populations, exact tests for population differentiation [55] as well as calculation of pair-wise estimates of *F*_ST_ [47] were performed using GENEPOP. The 95% significance levels for pairwise comparisons were adjusted using a sequential Bonferroni correction.

Isolation by distance (IBD) among geographic populations was tested using the Mantel test. The KJ populations sampled in 2005 (KJ05-S, KJ05-D) were again omitted from this analysis. The IBD analysis was carried out using two matrices, genetic distance (*F*_ST_) and geographic surface distance (in kilometers), in GENALEX v6.5 [43]. Geographic surface distance was calculated as the shortest distance between sampled populations via water (derived oceanographic distance) from the website (http://www.movable-type.co.uk/scripts/latlong.html). Geographic surface distance for depth populations within the three sites (KJ15, JD, AG) in the South Sea was considered as zero kilometers because of the close surface distances (within approximately 100 m) between them. To analyze IBD in smaller geographic scales, we performed two independent Mantel tests separately for populations in Jeju Island and those in the South Sea.

In addition, we analyzed the population structure of *Z*. *marina* using an individual-based Bayesian population assignment test in STRUCTURE v2.3.1 under a model of admixed ancestry among populations and correlated allele frequencies [56] with no a priori information on the geographic origins of the samples. STRUCTURE calculates a likelihood score when the data are forced into a given number of genetic clusters, *K*. We tested 10 iterations at each *K* = 1−14, with 50000 burn-in steps followed by 500000 Markov chain Monte Carlo (MCMC) generations. STRUCTURE analyses were also performed separately for populations in Jeju Island and for those in the South Sea. The “temporal” structure between the samples of KJ (KJ05-S, KJ05-D, KJ15-S, and KJ15-D) was examined at each *K* = 1−4. For this temporal comparison, depth samples were not pooled because statistically significant differentiation was observed between the 2015 samples (KJ15-S vs KJ15-D; *F*_ST_ = 0.031, *P* < 0.01). The most probable number of clusters (*K* value) was estimated using the ΔK method [57] implemented in the web-based tool, Structure Harvester (http://taylor0.biology.ucla.edu/structureHarvester), on the basis of the rate of change in the log probability of data between successive *K* values [58]. In addition, the number of genetic clusters (populations) was set to *K* = 11 (after excluding KJ05 samples), based on the results of significant pairwise *F*_ST_-statistics tested. We ran the analysis three independent times to check for convergence on similar *K* values, and found that the three runs arrived at identical values. Finally, genetic relationships among individuals with multilocus genotypes were assessed by factorial correspondence analysis (FCA) as implemented in GENETIX v4.04 [59].

## Results

### Clonal and genetic diversities

**Fig 2:**
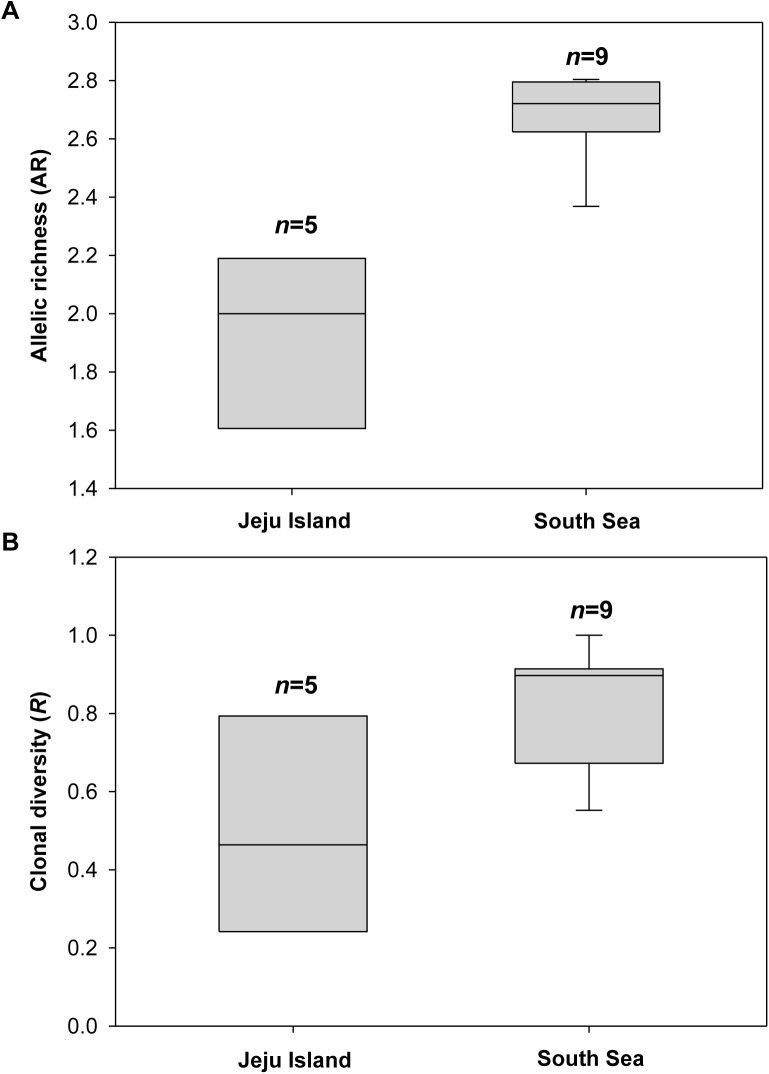
**Box plots illustrating differences in the level of genetic diversities such as allelic richness (A) and clonal diversity (B) between populations (*n* = 5) in Jeju Island and those (*n* = 9) in the South Sea.** The differences were statistically significant (allelic richness: Mann-Whitney *U* = 0, *P* = 0.001; clonal diversity: Mann-Whitney *U* = 7.5, *P* = 0.042). The thin line within the box is the median; the box marks the 25^th^ and 75^th^ percentiles; the whiskers mark the 10^th^ and 90^th^ percentiles.

Genetic diversity indices [e.g., clonal diversity (*R*), allelic richness (AR), mean number of alleles per locus (*N*_A_), heterozygosity] within the 16 populations of *Z*. *marina* on the Korean coasts are summarized in Table 1. The overall values of *P*_ID_ and *P*_IDsib_ were 0.0017 with a range of 0–0.0175 and 0.0436 with a range of 0.0074–0.2215, respectively, indicating a reasonable power to identify unique clones using our eight microsatellite markers and sample sizes [45]. The values of *P*_IDsib_ for the Jeju Island samples (mean = 0.0994) were generally higher than for the South Sea samples (mean = 0.0146) (Table 1), suggesting that values of *R* for the Jeju Island populations may be underestimated if near relative matings are common in these populations. Extents of within-population microsatellite diversity were significantly greater for the samples within the South Sea region than for those within the Jeju Island region (*R*: Mann-Whitney *U* = 7.5, *P* = 0.042; AR: Mann-Whitney *U* = 0, *P* = 0.001) (Table 1, Fig 2). Mean *R* for Jeju Island and the South Sea was 0.507 ± 0.288 (standard deviation; SD) and 0.822 ± 0.153, respectively. *N*_A_ for Jeju Island and South Sea ranged from 1.625 (HD) to 3.125 (SH) and from 3.750 (AG-D) to 5.250 (JD-S), respectively. As a consequence, the level of AR for Jeju Island was significantly lower than that for South Sea [Jeju Island: 1.524 (HD)– 2.255 (SH), mean = 1.918 ± 0.305; South Sea: 2.368 (KJ15-S)– 2.804 (AG-D), mean = 2.690 ± 0.142] (Table 1, Fig 2). Similarly, the observed (*H*_O_) and expected (*H*_E_) heterozygosity in Jeju Island [*H*_O_: 0.229 (HD)– 0.346 (TK), mean = 0.283; *H*_E_: 0.194 (HD)– 0.405 (SH), mean = 0.308] were much lower than those in the South Sea [*H*_O_: 0.380 (KJ15-S) − 0.625 (AG-D), mean = 0.473; *H*_E_: 0.404 (KJ15-S) − 0.556 (AG-D), mean = 0.483] (Table 1). Moreover, the number of private alleles detected was four times greater for the South Sea (*n* = 44) than for Jeju Island (*n* = 11). The KJ samples collected in 2015 showed a slightly higher level of AR than those collected in 2005 (mean AR for KJ15 = 2.582; KJ05 = 2.327; Table 1) as 12 alleles, which had not been present in KJ05 samples, were newly found in KJ15 samples.

The *F*_IS_ values within the Jeju Island and South Sea regions ranged from −0.198 (HD) to 0.251 (SH) and from −0.129 (AG-D) to 0.243 (KJ15-D), respectively. As a consequence, two populations (TK, and SH) on Jeju Island and three populations (JD-D, KJ15-D, and KJ05-D) in the South Sea might be experiencing non-random mating (e.g., inbreeding or outbreeding) at the eight loci analyzed, based on our multilocus tests for Hardy-Weinberg equilibrium (HWE) expectations (Table 1). In Jeju Island, TK showed a negative value for *F*_IS_, indicating outbreeding, but SH had positive values, indicating a significant deficiency of heterozygotes. In the South Sea, JD-D, KJ15-D and KJ05-D had positive values, ranging from 0.053 (JD-D) to 0.243 (KJ15-D). The estimated frequencies of null alleles at the eight loci were close to zero, ranging from 0.020 (GA1) to 0.1521 (CT20), suggesting a low probability of null alleles. Tests of LD between the eight loci were not significant after a sequential Bonferroni correction, suggesting that all of the loci analyzed can be considered independent markers.

BOTTELNECK analysis revealed that only two populations (HD and OJ) within Jeju Island and one population (KJ05-S) within the South Sea had allelic distribution shifts (mode-shift), which are typically regarded as evidence of population bottlenecks (S1 Table). However, heterozygosity excess was only detected in the two populations within Jeju Island. These two populations that showed allelic distribution shifts, however, displayed a statistically nonsignificant heterozygosity excess using a Wilcoxon sign rank test (S1 Table).

The LD method gave median estimates of effective population sizes (*N*_e_) of only 0.9 [95% confidence interval (CI): 0.6–1.5] and infinity (95% CI: 1.6 – ∞) for the WJ population and the OJ population on Jeju Island, respectively (Table 2). The estimates of *N*_e_ for the populations in the South Sea were, however, generally larger, ranging from 2.7 (95% CI: 1.7–7.9) for KJ05-D to an infinite *N*_e_ (95% CI: 50.1 – ∞) for JD-S (Table 2).

**Table 2:** Estimates of contemporary effective population sizes (*N*_e_) for 16 populations (including temporally replicated and depth samples) of *Zostera marina* based on linkage disequilibrium (LD) method using NeEstimator v2.01 [53]. Population abbreviations as in text and Fig 1.

POPULATION		Median of *N*_e_	95% confidence interval (CI)
**Jeju Island**	**HD**	1.5	0.5–27.5
	**WJ**	0.9	0.6–1.5
	**TK**	46.7	10.1 –infinity
	**SH**	21.1	5.4 –infinity
	**OJ**	infinity	1.6 –infinity
**South Sea**	**GM**	17.9	5.3–485.6
	**AG-S**	101.5	21.3 –infinity
	**AG-M**	17.2	8.3–59.2
	**AG-D**	29.6	10.7 –infinity
	**NP**	infinity	41.5 –infinity
	**JD-S**	infinity	50.1 –infinity
	**JD-D**	86.3	21.5 –infinity
	**KJ15-S**	997.2	22.8 –infinity
	**KJ15D**	29.5	15–105.7
	**KJ05-S**	9.6	3.1–37.7
	**KJ05-D**	2.7	1.7–7.9

### Population genetic structure

Spatial AMOVA analysis revealed significant variation in the genetic structure between the Jeju Island and South Sea regions (Table 3). In addition, significant genetic variation among populations within the regions was detected. However, the percentage of variation accounted for between the regions (22.11%) was higher than that for among populations within the regions (14.46%; Table 3). The overall genetic variation among populations, regardless of the groups, was also significant.

**Table 3:** Hierarchical analysis of molecular variance (AMOVA) of spatial genetic structure for the 14 populations of *Zostera marina* based on eight microsatellite loci. The KJ populations sampled in 2005 (KJ05-S, KJ05-D) were excluded from this analysis since detectable temporal genetic variation was observed. The analyses were performed by grouping the geographic populations according to the respective regions (Jeju Island and South Sea; see Materials and Methods).

Source of variation	d.f.	Variance component	% Variation	Fixation indices	*P*-value
Between Jeju Island and South Sea regions	1	0.609	22.111	*F*_CT_ = 0.221	0.000
Among populations within the regions	12	0.399	14.463	*F*_SC_ = 0.186	0.000
Within populations	564	1.748	63.427	*F*_ST_ = 0.366	0.000

Nearly all of the pairwise comparisons of *F*_ST_ statistics showed highly significant genetic differentiation among the 16 populations (except six comparisons between depth or temporal samples taken from the same sites: AG-S vs AG-M, AG-S vs AG-D, JD-S vs JD-D, KJ05-D vs KJ15-S, KJ05-D vs KJ15-D and KJ05-S vs KJ05-D), and *F*_ST_ values ranged from 0.009 (AG-S vs AG-M in the South Sea) to 0.573 (HD vs OJ in Jeju Island) (Table 4), indicating that limited gene flow has been occurring among populations. The degree of genetic differentiation was generally higher for the Jeju Island region (*F*_ST_ = 0.054 − 0.573) than for the South Sea region (*F*_ST_ = 0.005 − 0.346), suggesting that more restricted population connectivity was evident among populations in Jeju Island (Table 4).

**Table 4:** Spatial, temporal and depth-specific genetic differentiation between 16 populations of *Z*. *marina* in South Korea, based on eight microsatellite loci. The pairwise *F*_ST_ values between populations were calculated according to [47]. Nearly all of the pairwise *F*_ST_ values were statistically significant. Population abbreviations as in text and Fig 1. *: significant after a sequential Bonferroni correction applied (*P* < 0.05); **: *P* < 0.01.

	HD	WJ	TK	SH	OJ	GM	AG-S	AG-M	AG-D	NP	JD-S	JD-D	KJ15-S	KJ15-D	KJ05-S
**WJ**	0.531**														
**TK**	0.364**	0.374**													
**SH**	0.174**	0.293**	0.054**												
**OJ**	0.573**	0.386**	0.226**	0.208**											
**GM**	0.422**	0.282**	0.297**	0.289**	0.386**										
**AG-S**	0.418**	0.252**	0.240**	0.257**	0.339**	0.062**									
**AG-M**	0.465**	0.274**	0.252**	0.279**	0.324**	0.061**	0.009								
**AG-D**	0.470**	0.283**	0.277**	0.304**	0.336**	0.145**	0.025	0.036*							
**NP**	0.468**	0.268**	0.315**	0.315**	0.390**	0.077**	0.110**	0.116**	0.159**						
**JD-S**	0.535**	0.413**	0.415**	0.415**	0.488**	0.228**	0.237**	0.253**	0.236**	0.183**					
**JD-D**	0.538**	0.412**	0.445**	0.433**	0.507**	0.247**	0.263**	0.280**	0.260**	0.190**	0.013				
**KJ15-S**	0.517**	0.388**	0.399**	0.391**	0.500**	0.141**	0.119**	0.167**	0.193**	0.144**	0.320**	0.326**			
**KJ15-D**	0.457**	0.299**	0.360**	0.351**	0.450**	0.120**	0.108**	0.153**	0.152**	0.090**	0.182**	0.184**	0.031**		
**KJ05-S**	0.534**	0.370**	0.379**	0.367**	0.490**	0.137**	0.092**	0.135**	0.176**	0.131**	0.321**	0.333**	0.043**	0.069**	
**KJ05-D**	0.565**	0.418**	0.426**	0.410**	0.540**	0.161**	0.132**	0.178**	0.201**	0.160**	0.338**	0.346**	0.005	0.042	0.017

For depth samples at KJ, AG and JD in the South Sea, we found weak but some statistically significant genetic differentiation between shallow or middle and deep samples (Table 4). *F*_ST_ statistics between shallow and deep populations for the KJ05 samples showed no significant difference (*F*_ST_ = 0.017, *P* > 0.05). However, these populations after 10 years became significantly genetically divergent (KJ15: *F*_ST_ = 0.031, *P* < 0.01). Among samples from three depths (shallow, middle, deep) within the AG population, one population-pair was significantly genetically differentiated (AG-M vs AG-D, *F*_ST_ = 0.036, *P* < 0.05). There was no significant genetic differentiation detected between shallow and deep populations for JD (JD-S vs JD-D, *F*_ST_ = 0.013, *P* > 0.05) (Table 4). The pairwise estimates of *F*_ST_ statistics revealed a significant genetic divergence between the KJ05-S samples collected in 2005 and KJ15 samples in 2015 (Table 4).

**Fig 3:**
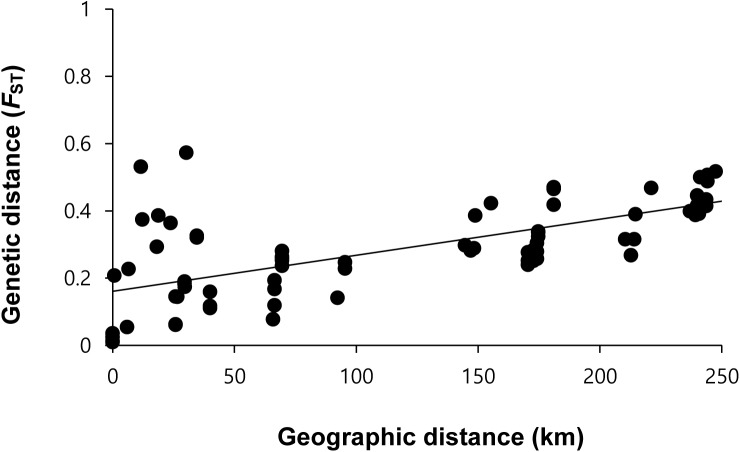
Results of isolation by distance (IBD) analysis with the Mantel test for the 14 populations of *Zostera marina* in South Korea. The KJ populations sampled in 2005 (KJ05-S, KJ05-D) were excluded from this analysis since temporal genetic variation was observed. We found a significant correlation between geographic and genetic distances across the populations (R^2^ = 0.455, *P* < 0.01).

The Mantel test showed a significant positive correlation between geographic (km) and genetic distances (*F*_ST_) for all 14 populations (R^2^ = 0.4549, *P* < 0.01) (Fig 3). Especially within the Jeju Island region, relatively high genetic distances between populations were detected, although those populations are geographically closely situated (e.g., HD vs WJ: 11 km, HD vs OJ: 30 km; Table 4). While the South Sea populations revealed a significant positive correlation between geographic and genetic distances (R^2^ = 0.3703, *P* < 0.01), Jeju Island populations showed a lack of a significant correlation between them (R^2^ = 0.0001, *P* = 0.33).

**Fig 4:**
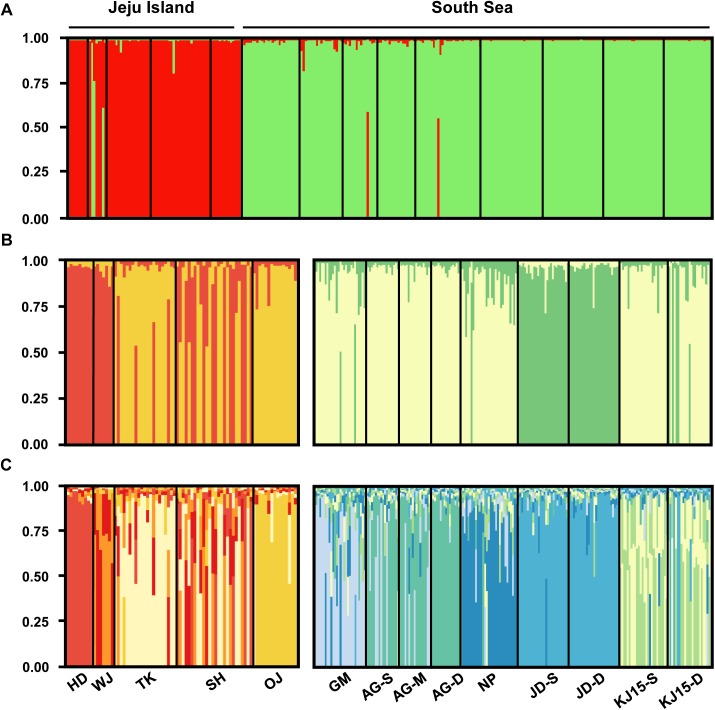
Analyses of spatial population structure using a Bayesian population assignment test with STRUCTURE, based on eight microsatellite loci. Each individual is represented along the x-axis, and the y-axis denotes the probability of that individual belonging to each of the genetic clusters. (A) Bar plot assuming 2 genetic clusters as suggested by STRUCTURE. (B) Left (Jeju Island): plot assuming 2 genetic clusters, right (South Sea): plot assuming 3 genetic clusters, as suggested by STRUCTURE. (C) Left (Jeju Island): plot assuming 5 genetic clusters, right (South Sea): plot assuming 6 genetic clusters, as suggested by pairwise *F*_ST_ statistics analyzed. Population abbreviations as in text and Fig 1.

**Fig 5:**
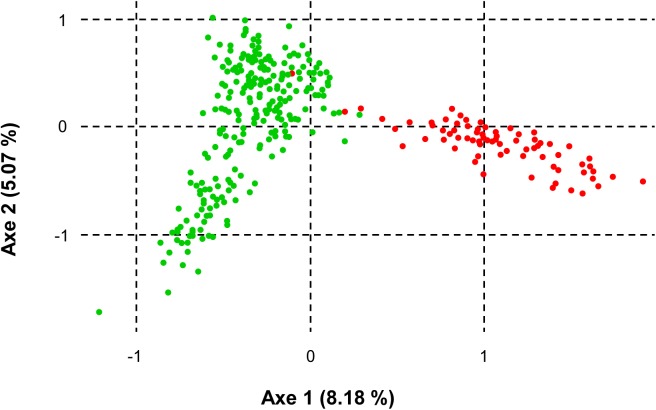
Factorial correspondence analysis (FCA) of eight microsatellite allelic variation for the 14 populations of *Zostera marina* in South Korea. Filled circles in red denote individuals of populations from Jeju Island, and those in green indicate individuals from the South Sea. Two genetic clusters were evident, which correspond well to the respective regions.

STRUCTURE analysis found that the 14 populations of *Z*. *marina* are most likely to form two genetically unique clusters (*K* = 2), which corresponds well to their geographical proximities (e.g., Jeju Island, South Sea) (Fig 4A). Two distinct genetic clusters, as for the Jeju Island and South Sea regions, were further supported by the results of our FCA (Fig 5). The number of genetic clusters was determined by statistics values, Δ*K* = 3674.59. Several individual samples from WJ on Jeju Island clustered with populations from the South Sea. When STRUCTURE analyses were performed separately for the two regions, populations from Jeju Island and the South Sea were shown to comprise two genetic clusters (HD and WJ; TK and OJ) with an admixed population (SH), and two clusters (GM, AG, NP and KJ; JD), respectively (Fig 4B). Genetic clusters as suggested by pairwise estimates of *F*_ST_ (Jeju Island: *K* = 5; South Sea: *K* = 6) are shown in Fig 4C. In Jeju Island, the WJ, TK and OJ populations had moderate level of a signature of genetic admixture, and the SH population showed high level, while the HD population was composed solely of a single genetic makeup. In the South Sea, seven populations (excluding JD-S and JD-D) examined possessed a signature of genetic admixture (Fig 4C).

Similar to the results of weak genetic differentiation between temporal samples for the KJ population (KJ05 and KJ15), STRUCTURE analysis suggested three genetic clusters (*K* = 3) that appeared to be distributed broadly across the depths and sampling years, perhaps due to low levels of genetic divergence between the samples (S1 Fig).

## Discussion

### Lower level of genetic diversity in Island populations

Seagrasses play a central role in the ecosystem functioning of coastal and estuarine habitats [60]. Seagrasses worldwide are, however, under severe threat due to sharp population declines in recent decades, and one in five seagrass species is now at risk of extinction [11, 12]. Natural recovery of perturbed seagrass meadow requires a considerable amount of time, even though seagrass die-off is rather rapid [61]. Therefore, seagrass conservation and restoration management efforts through transplantation are currently under way in many parts of the world, including Korea [36–38,62]. However, seagrass transplantation projects often focus on increasing the density, productivity, and area of meadow coverage, while information on the genetic diversity and genetic structure of source and recipient populations, which is suggested to be a key factor in the ultimate outcome of restoration efforts [63], is nonetheless often overlooked. Transplantation should also be accomplished without perturbing the natural genetic structure, because natural seagrass populations typically show spatial genetic structure, which is often apparently associated with particular local environments (e.g., oceanic current) [64] and/or spatially and temporally varying patch dynamics (e.g., ‘genetic patchiness’) [18,19]. Therefore, understanding the population genetic structure of seagrass species occurring at a given local environment should be an essential component of conservation and restoration efforts.

In the present study, we first reported on the levels of within-population genetic diversity and population genetic structure among the 16 populations (including temporal and depth samples) of the temperate seagrass species, *Z*. *marina*, in Jeju Island and the South Sea on the southern part of the Korean Peninsula using eight nuclear microsatellite loci. We found that populations from Jeju Island, which is located between the southernmost region of the Korean Peninsula and Japan where the water mass from the Kuroshio Current meets the Yellow Sea, serving as a biological hotspot in Korea [65], harbor significantly lower levels of within-population genetic diversity (e.g., AR, *R*) than those from the South Sea. The observed lower levels of genetic diversity in five populations from Jeju Island (mean AR = 1.92), which is located approximately 155 km off the mainland, relative to those from the South Sea (AR = 2.69), which is situated on the southern coast of the mainland, suggest that effective population sizes (*N*_e_) have been smaller for the former than for the latter. Multifaceted lines of evidence support the hypothesis that Jeju Island populations have a smaller current *N*_e_. Our microsatellite-based LD estimates of *N*_e_ indicate that a median of *N*_e_ at Jeju Island [mean *N*_e_ = 17.55 when OJ (*N*_e_ = infinity) was excluded] is approximately eight times smaller than that at the South Sea [mean *N*_e_ = 143.50 when NP and JD-S (*N*_e_ = infinity) was excluded], providing direct support for our hypothesis (Table 2). Two of the five populations from Jeju Island (HD and OJ) show a genetic signal of a “population bottleneck”, as suggested by the incidences of allele distribution shift or heterozygote excess, further supporting the hypothesis that these particular populations on Jeju Island have probably undergone a recent bottleneck [51].

Levels of within-population genetic diversity in plants are suggested to be positively associated with population size and fitness [66]. For eelgrass, a previous study experimentally demonstrated a positive correlation between a measure of genetic diversity as represented by the number of alleles per locus and individual survivorship and thus, population size during a 3-year period [26]. The observed trend of the positive relationship between genetic diversity and population size or coverage (HD = 138 m^2^, WJ = 310 m^2^, OJ = 841 m^2^, TK = 4438 m^2^, and SH = 275736 m^2^; S.R. Park, unpublished data) estimated from Jeju Island appears to be commensurate with those previous reports. The SH population, which has the largest meadow area, shows the highest level of genetic diversity (AR = 2.26) whereas the HD population, which has the smallest area, shows the lowest level of genetic diversity (AR = 1.52). Although the WJ population’s coverage is approximately half that of OJ, the level of genetic diversity in WJ (AR = 2.12) is even higher than in OJ (AR = 1.69). This unexpected, relatively high genetic diversity in WJ is possibly due to a human-mediated transplantation project undertaken using other populations as a source material around this region in 2009 [39]. Interestingly, the level of genetic diversity in the KJ population in the South Sea has slightly increased over the last decade, implying that *N*_e_ has been augmented in this population, which is supported by our estimates of *N*_e_ (Table 2). The patterns of elevated levels of genetic diversity in KJ in 2015 relative to 2005 are apparent in both shallow and deep populations. The increased genetic diversity in KJ over those 10 years may contribute to the observed temporal genetic structure between KJ05 and KJ15 samples (see below).

Geographically disconnected and genetically isolated populations with a relatively smaller *N*_e_ on Jeju Island are more vulnerable to the effects of increased genetic drift causing the loss of rare alleles [67]. This diminishes the evolutionary potential of the population to genetically adapt to novel environmental conditions such as climate change [68]. This is particularly critical, given that populations from Jeju Island are situated off the southern end of the Korean Peninsula, which is the warmer area and is classified as subtropical weather zone recently; this region is also one of the fastest warming regions worldwide [69]. Therefore, the Jeju Island populations should be conserved with high priority. According to a previous study [25], a high extent of genetic diversity may enhance ecosystem recovery, such as biomass production, plant density, and faunal abundance, after perturbations. Loss of genetic diversity resulting from the increased effects of inbreeding and genetic drift may elevate the probability of extinction of small populations [70]. In this respect, Jeju Island populations are perhaps at high risk of local extinction under changing environments, as they have not only a low degree of genetic diversity (AR, *R*) but also small *N*_e_.

### Strong spatial but weak temporal genetic structure

We find that the eelgrass, *Z*. *marina* on the southern coast of the Korean Peninsula comprises genetically divergent populations on a 0.7–250 km geographic scale at a given time. Our multifaceted analyses of a microsatellite dataset clearly reveal a noticeable level of genetic structure between populations in Jeju Island and those in the South Sea, as suggested by spatial AMOVA as well as an individual based Bayesian population assignment test and also FCA analyses. The observed significant genetic structuring between the Jeju Island and South Sea regions suggests a very low level of ongoing gene flow occurring between populations across the South Sea of Korea. Even within each region, microsatellite genetic differentiation was fairly high (Jeju Island: *F*_ST_ = 0.054 − 0.573; South Sea: *F*_ST_ = 0.009–0.326; Table 4) and highly statistically significant (except some population comparisons between depth or temporal samples). The magnitude of genetic differentiation is, however, generally higher for populations in Jeju Island than for those in the South Sea, although the former localities (approximately 0.7 − 30 km) are geographically closer to each other than the latter (approximately 26 − 95 km). Isolation by distance (IBD) analysis further indicates that genetic distance among populations increases with geographic distance, meaning that the geographic proximity of populations contributes to shaping the observed spatial genetic structure. However, geographic distance cannot explain the genetic variation observed among populations in Jeju Island.

Most, but not all, other population genetics studies on spatial genetic variation in the eelgrass *Z*. *marina* have also found genetic heterogeneity over rather small geographic scales of a few or tens of kilometers [28,71]. This can be interpreted as restricted ongoing gene flow taking place among geographically disconnected populations of *Z*. *marina*. At even smaller, fine-grained scales, a mosaic of clones that originate from different source populations over space and time can also lead to the micro-geographical population structure in this species [18,19]. There appears to be two possible modes of dispersal across populations of *Z*. *marina* [21]: (1) short- distance dispersal of seeds from a nearby parental population by gravity [72] and (2) long- distance dispersal from a far-away parental population by rafting of shoots containing seeds over several tens of kilometers [73] and even sometimes up to a few hundred kilometers via local oceanic currents [74,75]. In this regard, the geographic distance between HD and WJ on Jeju Island is approximately 11 km, but the degree of genetic differentiation is the second highest among populations on Jeju Island (*F*_ST_ = 0.531), suggesting that dispersal via rafting seems highly unlikely in this region, perhaps due to local currents. The restricted population connectivity, particularly among Jeju Island populations might, at least in part, explain why levels of within-population genetic diversity on Jeju Island are generally lower than in the South Sea, and this may ultimately lead to a reduction in population fitness, thereby elevating the risk of local extinction. On the other hand, directional selection can cause a reduction in genetic diversity rapidly, although it increases average population fitness. Yet, this scenario seems unlikely, given presumed selective neutrality of microsatellite markers used in this study.

We also observed a statistically significant change in population structure between temporal samples (2005 vs 2015) in a shallow population of KJ in the South Sea (Table 4). Twelve alleles that were not present at KJ in 2005 were newly detected at the same sites after 10 years had elapsed. In addition, the frequencies of 10 and 13 alleles for shallow and deep populations, respectively, at KJ have changed over the last decade. Therefore, the level of genetic diversity in KJ15 (mean AR = 2.582) is higher than in KJ05 (mean AR = 2.327). In this area, the *Z*. *marina* population was maintained through only asexual reproduction (new shoot recruitment via lateral shoot production) before 2006; however, seedling shoots via sexual reproduction have been observed since 2006, and their density has gradually increased in both shallow and deep zones (S.R. Park, personal observation). The observed weak temporal variation in the genetic structure can also be explained by the temporally varying clonal dynamics and genetic mosaic of eelgrasses [19]. Our data and observations, however, suggest that the observed new alleles have come into KJ from other South Sea regions (e.g., GM, AG and JD) by floating seed dispersal over 10 years and thereby, changed its genetic composition.

### Genetic divergence by water depth

The life history or reproductive strategy of *Z*. *marina* is known to be affected by various environmental parameters such as different light regimes [29,76], salinity fluctuations, and water temperature [77]. According to a previous study [29], a deep population (water depth ranges from 4 to 7 m) at JD in the South Sea maintained its meadow only through sexual reproduction (typical annual life cycle) whereas a shallow population (water depth ranges from 1 to 3 m) at the same site persisted through both sexual and asexual reproduction (typical perennial life cycle).

We find weak but statistically significant genetic differentiation between shallow (and middle) and deep populations of *Z*. *marina* at two (AG and KJ) of the three localities in the South Sea region analyzed (Table 4). The results may suggest that life history differences between shallow and deep populations [29] are hindering genetic exchange to some detectable degree along the depth gradient in these particular environments. Light attenuation along the depth may act as a barrier to gene flow between shallow and deep populations examined [29]. However, this hypothesis should await future ecological investigation. Alternatively, other environmental factors, such as tidal changes, may serve as a barrier to genetic exchange, leading to the observed genetic divergence among samples from different depths. In addition, differences in the disturbance regime along the depth gradient may lead to differences in the frequencies of an opportunity for subsequent (chaotic) recolonization, causing the fine-grained population structure [18]. A previous study found only marginally significant genetic differentiation between shallow and deep populations at one of the three sites tested in San Francisco Bay, California, USA [28]. Additional population genetic studies using samples taken from various depths at other localities would be required to generalize the observed patterns of genetic divergence along the depth gradient.

### Conservation implications

Knowledge about the population genetics of seagrass is an important issue for its effective conservation and restoration management [15,25,28]. Multiple lines of evidence–depauperate within-population genetic diversity, small *N*_e_, and limited levels of genetic exchange among populations on Jeju Island–suggest an urgent need to conserve these vulnerable populations. Other populations with higher levels of genetic diversity could be used as source materials for restoration of the eelgrass populations through transplantation [78]. However, the possibility of disrupting localized adaptation that may already be present in the recipient population should also be considered. For *Posidonia oceanica*, a seagrass species endemic to the Mediterranean Sea, enriched genetic diversity of source populations was significantly positively correlated with individual survival, increased rhizome length (i.e., growth), and number of ramets in the transplanted shoots [79]. However, transplantation should be undertaken carefully, considering not only genetic diversity but also the “natural” spatial population structure that is possibly related to particular local environments [64].

## Conclusions

This study first provides the information on the genetic diversity and genetic structure of South Korean eelgrass (*Zostera marina*) populations, which will contribute to the establishment of appropriate management, conservation, and restoration plans for future persistence of this ecologically valuable species. We genotyped eight microsatellite loci for 454 individuals sampled from 16 populations (including temporally replicated and depth samples) along the southern coastal regions of the Korean Peninsula (e.g., Jeju Island and South Sea). We found significantly lower levels of genetic diversity, smaller *N*_e_ and more restricted population connectivity (i.e., contemporary gene flow among populations) for Jeju Island compared with the mainland populations, suggesting that the southernmost populations off the Korean Peninsula are more vulnerable to local extinction under future changing environments. We suggest that the Jeju Island eelgrass populations should be conserved with high priority, given that this region is known to harbor the highest level of biodiversity in spite of being one of the fastest warming regions around the world. Further studies of *Z*. *marina* along the western and eastern coasts of the Korean Peninsula would help us to better understand the broader pattern of population genetic structure of Korean eelgrass and also to develop effective restoration strategies for this species.

## Supporting information

S1 Fig Analyses of temporal population structure using a Bayesian population assignment test with STRUCTURE, based on eight microsatellite loci. (DOCX)

S1 Table Statistical tests for a recent bottleneck in each of the 16 populations (including temporally replicated and depth samples) of *Zostera marina* from South Korea. (DOCX)
