# Supplementary material for: Population genetic structure of eelgrass (Zostera marina) on the Korean coast: Current status and conservation implications for future management
Source: PLoS One. 2017 Mar 21;12(3):e0174105. doi: 10.1371/journal.pone.0174105 (PMC5360257; doi:10.1371/journal.pone.0174105)
Supplement: S1 Fig — (DOCX) [file pone.0174105.s001.docx]

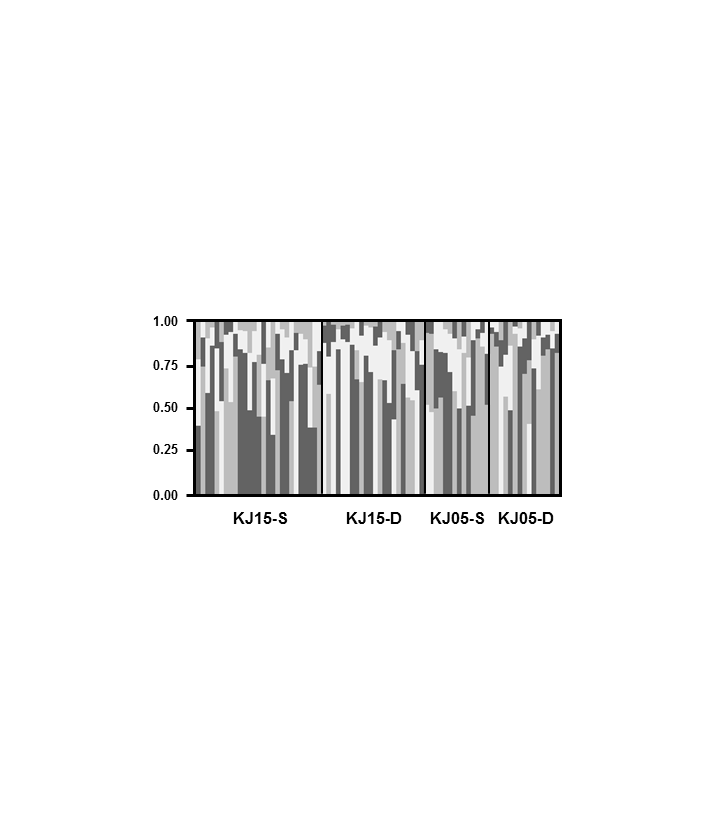


**S1 Fig. Analyses of temporal population structure using** **a** **Bayesian population assignment test with STRUCTURE, based on eight microsatellite loci.** Plot assuming 3 genetic clusters (*K* = 3) supported by STRUCTURE. KJ05: samples collected at KJ in 2005; KJ15: samples obtained at KJ in 2015. –S: shallow zone, –D: deep zone.
