# Supplementary material for: Population genetic structure of eelgrass (Zostera marina) on the Korean coast: Current status and conservation implications for future management
Source: PLoS One. 2017 Mar 21;12(3):e0174105. doi: 10.1371/journal.pone.0174105 (PMC5360257; doi:10.1371/journal.pone.0174105)
Supplement: S1 Table — (DOCX) [file pone.0174105.s002.docx]

**S1 Table. Statistical tests for a recent bottleneck in each of the 16 populations (including temporally replicated and depth samples) of *Zostera marina* from South Korea.** *P*-values are based on the Wilcoxon’s statistical tests. Allelic frequency distribution shape was normal or shifted for mode-shift distortion. Monomorphic loci were excluded in these analyses.

| **POPULATION** | | **deficiency** | **excess** | ***P*-value** | **mode shift** |
| --- | --- | --- | --- | --- | --- |
| **Jeju Island** | **HD** | 2 | 3 | 0.813 | shifted |
|  | **WJ** | 5 | 3 | 0.055 | normal |
|  | **TK** | 2 | 3 | 0.844 | normal |
|  | **SH** | 4 | 4 | 0.844 | normal |
|  | **OJ** | 2 | 4 | 0.563 | shifted |
| **South Sea** | **GM** | 5 | 3 | 0.742 | normal |
|  | **AG-S** | 3 | 5 | 0.844 | normal |
|  | **AG-M** | 4 | 4 | 0.844 | normal |
|  | **AG-D** | 1 | 7 | 0.055 | normal |
|  | **NP** | 6 | 2 | 0.250 | normal |
|  | **JD-S** | 6 | 2 | 0.074 | normal |
|  | **JD-D** | 4 | 4 | 0.383 | normal |
|  | **KJ15-S** | 5 | 3 | 0.195 | normal |
|  | **KJ15-D** | 7 | 1 | 0.027 | normal |
|  | **KJ05-S** | 5 | 3 | 0.383 | shifted |
|  | **KJ05-D** | 7 | 1 | 0.020 | normal |
